# Risk factors in non‑surviving patients with infection with carbapenemase‑producing Enterobacterales strains in an intensive care unit

**DOI:** 10.3892/mi.2023.90

**Published:** 2023-06-14

**Authors:** Nicoleta-Dorina Vlad, Elena Dumea, Claudia-Simona Cambrea, Cristina Gabriela Puscasu, Constantin Ionescu, Bianca Averian, Raluca-Vasilica Mihai, Andrei Dumitru, Irina-Magdalena Dumitru

**Affiliations:** 1 Clinical Infectious Diseases Hospital of Constanta, 900709 Constan£a, Romania; 2 Military Emergency Hospital Constanta, 900228 Constan£a, Romania; 3 Doctoral School of Medicine, Ovidius University of Constan£a, 900470 Constanta, Romania; 4 Faculty of Medicine, Ovidius University of Constan£a, 900470 Constanta, Romania; 5 Faculty of Dentistry, Ovidius University of Constan£a, 900470 Constanta, Romania; 6 Romania Academy of Sciences, 50085 Bucharest, Romania

**Keywords:** risk factors, mortality, carbapenemase-producing Enterobacterales, intensive care unit, COVID-19

## Abstract

Carbapenemase-producing Enterobacterales (CPE) are Gram-negative bacteria that belong to the Enterobacterales family and produce enzymes known as carbapenemases, which inhibit carbapenems, cephalosporins and penicillins. Carbapenem-resistant Enterobacterales (CRE) are resistant to carbapenems, cephalosporins and penicillins via mechanisms that may or may not produce carbapenemases. The identification of carbapenems is critical for the initiation of proper antibiotic therapy. The present case-control, retrospective study included 64 patients with CPE strains admitted to an intensive care unit between September, 2017 and October, 2021; of these, 34 patients with CPE succumbed and 30 control patients with CPE strains survived. CPE strains in the deceased patients were caused by *Klebsiella* spp. in 31 cases (91.2%) and *Escherichia coli* in 3 cases (8.8%). The univariate analysis revealed that the predictive factors associated with mortality in patients with CPE were admission with coronavirus disease 2019 (COVID-19) (P=0.001), invasive mechanical ventilation (P=0.001), and treatment with corticosteroids (P=0.006). The multivariate analysis revealed that admission with COVID-19 [odds ratio (OR), 16.26; 95% confidence interval (CI), 3.56-74.14; P≤0.05] and invasive mechanical ventilation (OR, 14.98; 95% CI, 1.35-166.22; P≤0.05) were associated with mortality as independent risk factors. Admission with COVID-19 increased the risk of mortality 16.26-fold and invasive mechanical ventilation increased the risk of mortality by 14.98-fold. On the whole, the present study demonstrates that the length of hospital duration in patients who acquired CPE did not influence mortality, whereas infection with COVID-19 increased and invasive mechanical ventilation were associated with an increased risk of mortality.

## Introduction

As early as 2010, there were concerns about the spread of carbapenemase-producing Enterobacterales (CPE) in healthcare facilities in Europe; therefore, the European Union (EU) member states proposed an assessment of the risks of spreading CPE from one patient to another (1).

Although the prevalence of CPE within the EU community is not well known, it is acknowledged that these strains are endemic to certain countries, including Romania (1). Carbapenemases are β-lactamases that efficiently hydrolyze most β-lactams, including carbapenems (1). CPE and carbapenem-resistant Enterobacterales (CRE) strains usually include *Escherichia coli* and *Klebsiella pneumoniae* (1,2).

The European Center for Disease Prevention and Control (ECDC) claims that CRE is associated with a high mortality rate; thus, it is considered that patients infected with CPE also have a high risk of mortality. The ECDC also affirms that the causes of death are due to the limitations of treatment options or due to delays (1).

The risk factors associated with CPE infection are similar to those associated with other multidrug-resistant organisms (MDRO) (1,2). The risk factors for acquiring CPE are prior antimicrobial use, previous hospitalization, admission to the intensive care unit (ICU), the severity of illness, a duration of hospitalization >20 days, the use of antibiotics for >10 days, pneumonia or chronic pulmonary disease, and the previous use of nasogastric tubes (1,3-5).

CPE infections represent a threat to patient safety owing to antimicrobial resistance (AMR), increased morbidity and mortality, and very high hospital costs (1). The ECDC claims that in the European Union/European Economic Area (EU/EEA), 33,000 individuals succumb each year as a direct consequence of MDRO infections, and >670,000 infections are caused by these organisms (6). According to the Centers for Disease Control and Prevention (CDC) in the USA, it has been estimated that the direct healthcare costs associated with AMR is up to 20 billion dollars (7). Otter *et al* (8) published an article on 40 patients from five hospitals and found that CPE outbreaks were associated with high costs. A study from 2003 to 2017 carried out in Scotland, in which 290 CPE strains from clinical and long-term healthcare surveillance cultures were detected, supported the fact that an age >60 years, systemic infection or organ failure, and the presence of non-fermenters were independently associated with 30-day mortality (9).

In another study published by Pintado *et al* (10), patients with coronavirus disease 2019 (COVID-19) were observed to have an increased risk of CPE infections and were associated with a high risk of mortality. Following a systematic literature review performed by searching the EMBASE, PubMed, and Cochrane Library databases, Hu *et al* (11) found that the risk factors most frequently associated with CRE mortality were antibiotic use, comorbidities and hospital-related factors (11).

The available data on the mortality of patients with CPE and the associated risk factors in Romania are not sufficient. International data identified through the European Antimicrobial Resistance Surveillance Network (EARS Net) support the fact that in Romania, the rates of CRE are increased, and the levels of AMR in Romania are a matter of concern (6,12).

The present study aimed to identify CPE strains and assess the risk factors for mortality in patients with CPE strains who were hospitalized in the ICU.

## Patients and methods

### Study design

A retrospective, case-control study was conducted in a single center, which included patients confirmed to be infected wiht CPE between September, 2017 and October, 2021, who were admitted to the ICU of the Infectious Diseases Hospital of Constanta, Romania. The inclusion criterion was an ICU admission >24 h associated with CPE infection or colonization. The exclusion criteria were non-admission to the ICU and an ICU admission of <24 h. CPE strains were detected upon admission to the ICU or throughout the course of admission. Bacteriological screening for bacterial colonization with CPE was performed upon admission to the ICU and every 7 days after hospitalization. CPE strains were found in rectal swabs, urine cultures, sputum, blood cultures and skin swabs. Demographic, epidemiological, clinical, paraclinical and treatment data were analyzed. The cause of hospitalization in the ICU was predominantly the severity of COVID-19 infection, and to a lesser extent, the CPE infection. It should be reported that CPE colonization was detected only in the ICU, which is the only department in the hospital where bacteriological screening was performed to detect these organisms.

**Figure 1:**
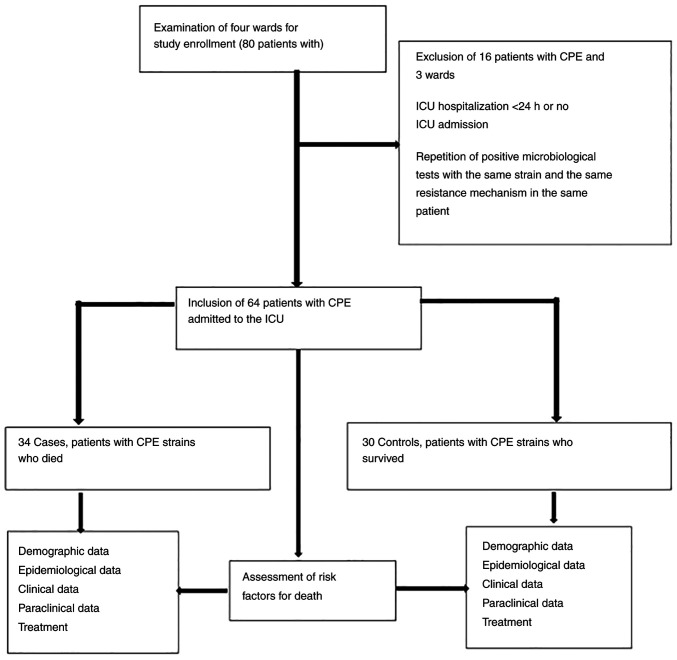
Schematic diagram of the design of the present study. CPE, carbapenemase-producing Enterobacterales; ICU, intensive care unit.

The study population was divided into cases (patients with CPE who succumbed) and controls (patients with CPE who survived). The ICU of the Infectious Diseases Hospital of Constanta included 10 beds for critical patients with infections. The data were collected from the IT system of the hospital. Demographic, epidemiological, clinical and paraclinical data, and patient treatment data were analyzed. These data included variables such as sex, age, the Charlson Comorbidity Index, admission with COVID-19 or bacterial infection, previous hospitalization or previous antibiotic therapy, leukopenia upon admission, C-reactive protein (CRP) levels >5 mg/l upon admission, invasive mechanical ventilation, CPE colonization or CPE infection, and length of stay (LOS) in the ICU. The inclusion and exclusion criteria, and the data used for analysis are presented in Fig. 1.

### Microbiology

In the Infectious Diseases Hospital of Constanta ICU, 73 CPE strains were detected in patients hospitalized for >24 h. There were 52 cases of CPE colonization and 21 cases of CPE infection in 64 patients. CPE colonization strains were detected by bacteriological screening using carbapenem-resistant Enterobacteriaceae chromogenic media and the Modified Hodge test. CPE strains were detected using the VITEK 2 system (bioMérieux) or matrix-assisted laser desorption/ionization time-of-flight mass spectrometry. Rosco discs and the Modified Hodge test confirmed carbapenemase production. Infection with severe acute respiratory syndrome coronavirus 2 (SARS-CoV-2) was detected using the RT-PCR SARS-CoV-2 test. The strains were identified in the blood, sputum, urine and rectal swab tests or skin swabs. CPE strains, including *Klebsiella* spp. and *Escherichia coli* were identified. Colonization was distinguished from infection by an infectious disease doctor according to the patient's clinical and paraclinical criteria. A total of 27 CPE infections were detected according to the clinical and paraclinical data interpreted by the infectious disease physician. All strains detected in the rectal swab through the bacteriological screening program carried out in the ICU of the hospital were bacterial colonization and not infections. Other cultures performed at the time of admission or during hospitalization at the indication of the attending physician were interpreted as colonization or infection. Patients with colonization did not meet the criteria for inflammation and had no signs or symptoms, whereas patients with an infection had multiple signs and symptoms that were specific to an infectious disease. The antibiogram was performed only for the isolates detected in the urinary tract, sputum and blood samples, and it was not performed for the rectal swab cultures, as recommended by the hospital in terms of costs. The antibiogram was interpreted according to the guidelines of the European Committee for Antimicrobial Susceptibility Testing (EUCAST).

### Ethical review and approval

The present study was conducted in accordance with the principles of the Declaration of Helsinki (13). All patients provided written informed consent for the use of their personal data upon hospital admission. Patient anonymity was guaranteed during the whole process of data analysis and reporting of results. The Infectious Diseases Hospital of Constanta considered ethical review and approval unnecessary due to the retrospective nature of the study (NR 1/20/01.2023; CODE F.05.PO.17.00-ACFOCG). This study conformed to the Strengthening the Reporting of Observational Studies in Epidemiology (STROBE) guidelines (14).

### Statistical analysis

Statistical analysis was performed using IBM SPSS Statistics, version 20.0 (IBM Corp.), and the data collected were imported into Microsoft Excel. The study was divided into two groups as follows: Cases (patients with CPE strains who succumbed) and controls (patients with CPE strains who survived). The variables associated with mortality with values of P≤0.05 in the univariate analysis were evaluated using the χ^2^ test. Variables with values of P≤0.05 in the univariate analysis were selected for logistic regression analysis as a multivariate statistical method. The hypothesis testing was two-tailed, and a value of P≤0.05 was considered to indicate a statistically significant difference.

## Results

According to the inclusion and exclusion criteria, 64 patients with CPE were admitted to the ICU, including 34 cases (patients with CPE who succumbed) and 30 controls (patients with CPE who survived). As demonstrated in to Table I, it was found that *Klebsiella* spp*.* strains were predominant, totaling 53 strains of which 31 were identified in patients who succumbed (with a 91.2% prevalence, out of 34 strains). *Escherichia coli* was detected in 3 patients (8.8%) who succumbed and in 8 patients (26.7%) who survived. In the univariate analysis, this strain in the patients did not exhibit a statistically significant difference (P>0.05). As regards the isolates detected in patients with CPE who succumbed, 27 strains (79.4%) were identified in rectal swab samples, two strains (5.9%) were identified in urine culture, eight strains (23.5%) were identified in sputum and three strains (8.8%) were identified in blood samples. Univariate analysis revealed that the source of the CPE strains was not statistically significant (P>0.05) (Table I).

**Figure 2:**
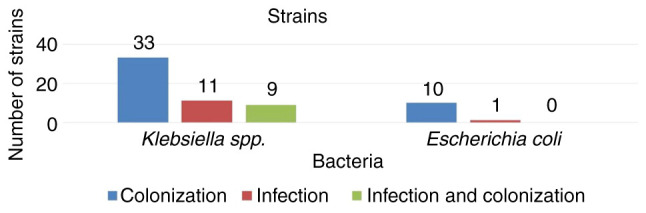
The number of CPE colonizations, infections, or both with *Klebsiella* spp., and CPE colonization and infections with *Escherichia coli*. CPE, carbapenemase-producing Enterobacterales.

As demonstrated in Fig. 2, it was observed that the majority of patients with bacterial colonies were detected in the ICU. Of the 64 patients, 53 (82.8%) were with *Klebsiella spp*. Of the 53 patients with *Klebsiella* spp., 44 (83.01%) had only carbapenemase-producing *Klebsiella spp*., of which 33 (75%) had colonization and 11 (25%) had bacterial infections. Of the 53 patients with *Klebsiella* spp., 9 patients (17%) had bacterial colonization and infection with the same carbapenenase-producing *Klebsiella* spp. strain.

**Figure 3:**
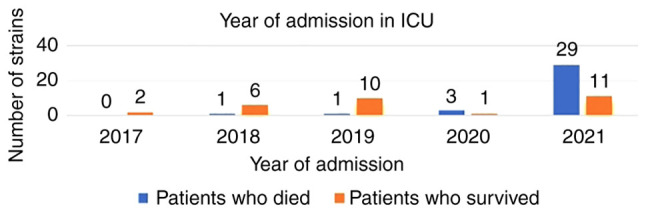
The number of patients with carbapenemase-producing Enterobacterales infections who succumbed or survived. ICU, intensive care unit.

It was also observed that the majority of patients passed away in 2021, while in 2017, all the patients with CPE strains survived (Fig. 3).

The medical history and demographic, clinical and paraclinical data of the patients are presented in Table II. Of the total number of patients who succumbed in the ICU, 24 were male (70.6%), while of those who survived, 21 were male (70%). The median age of the deceased patients was 63.88, while the median age of those who survived was 64.67. Among those who succumbed, 19 patients (55.9%) were >65 years of age. As regards comorbidities, considering a Charlson Comorbidity Index (CCI) score ≥4, the findings were similar in both groups, with 18 patients (52.9%) in the case group and 16 (53.3%) in the control group.

Other possible predictors for mortality may be admission with COVID-19 or admission with a bacterial infection. It was determined that the number of patients with COVID-19 was 30 (88.2%) in the deceased group, and 8 (26.7%) in the control group. Admission with bacterial infection was predominant in the control group, with 17 cases (56.7%) among the patients who survived, compared to 5 cases (14.7%) among the patients who succumbed. Upon univariate analysis, a statistically significant positive association was noted between admission with COVID-19 and mortality [odds ratio (OR), 20.62; 95% confidence interval (CI), 5.50-77.23; P=0.001].

As regards the epidemiological data, there were 7 patients (20.6%) with previous hospitalization who succumbed and 13 patients with previous hospitalization (43.3%) in the group of patients who survived. Another observation was that there were 22 patients (64.7%) who had received previous antibiotic therapy among the group of patients who succumbed during hospitalization in the ICU and 14 patients (46.7%) who had received previous antibiotic treatment among those who survived.

For the paraclinical data, 26 patients (76.5%) with leukopenia upon admission to the ICU succumbed, whereas 20 patients (66.7%) had leukopenia in the group of patients who survived. A CRP level >5 mg/l upon admission to the ICU was identified in 33 patients (97.1%) who succumbed compared to 26 patients (86.7%) who survived. It should be specified that the patients who were mechanically ventilated had a high mortality rate, compared to the patients who were not mechanically ventilated. Specifically, 13 patients (38.2%) who were mechanically ventilated succumbed, whereas among the surviving patients, only 1 patient (3.3%) was mechanically ventilated. Furthermore, univariate analysis confirmed a statistically significant positive association between invasive mechanical ventilation and mortality (OR,17.95; 95% CI, 2.17-148.08; P=0.001).

As regard the LOS in the ICU, as shown in Table II, it was found that there were 29 patients (85.3%) who had a LOS in the ICU >3 days after the CPE diagnosis among those who succumbed, while the number of patients with a LOS >3 days in the ICU among the group of patients who survived was 24 (80%). The univariate analysis of LOS >3 days after the diagnosis of CPE revealed no significant association with mortality (P>0.05).

No statistically significant differences were observed for the patient mortality and survival rates in patients with CPE colonization on the one hand, and patients with CPE infection on the other hand.

As regards the treatment prescribed to patients who were admitted to the ICU, it was observed that all patients who succumbed had received antibiotic treatment, and 26 (76.5%) of them had received reserve antibiotics. Of the 6 patients who received ceftazidime-avibactam treatment, 2 (5.9%) were in the group of patients who succumbed, and 4 (13.3%) were in the group of patients who survived. For treatment with other new antibiotics, 1 patient from the group who succumbed (2.94%) received ceftolozane-tazobactam, and 1 patient from the control group (3.3%) received imipenem-cilastatin-relebactam. Colistin was administered to 5 patients (14.7%) who succumbed and to 2 patients (6.7%) who survived. Carbapenem treatment was administered to 17 patients (50%) who died and eight patients (26.7%) survived. Other treatments, such as oral vancomycin were predominantly administered to patients who survived (OR, 0.02; 95% CI, 0.12, 0.01-0.97; P=0.025), while corticosteroid treatment was administered predominantly to patients who succumbed (OR, 4.66; 95% CI, 1.49-14.52; P=0.006). The treatment data of the patients are presented in Table III.

Multivariate analysis revealed that admission with COVID-19 and invasive mechanical ventilation were independent risk factors for mortality in patients with CPE. The statistical data are presented in Table IV.

## Discussion

In the present study, as in other studies, SARS-CoV-2 infection and invasive mechanical ventilation were determined to increase the risk of mortality in patients admitted to the ICU (10,15,16). Contou *et al* (17) claimed that patients with COVID-19 had a high mortality rate, particularly if they were intubated. In their study, they referred to a large multicenter study in which it was reported that 14% of patients with SARS-CoV-2 infection who were in critical condition experienced cardiac arrest (17). In another study, multivariate analysis revealed that the APACHE II score, age, the need for invasive mechanical ventilation, increased creatinine levels, and decreased serum albumin levels were independent risk factors for mortality (18). In their study, Zhao *et al* (9) reported that an age >60 years, the presence of non-fermenters and systemic infection or organ failure were independently associated with 30-day mortality in patients with CPE infection.

Although there are studies that have reported an increased risk of mortality in patients with CPE infection (19,20), this was not demonstrated in the present study. In previous research, multivariate analysis revealed that mechanical ventilation and the presence of indwelling medical devices were significant risk factors for mortality in patients with CPE infection (21).

As regards LOS in the ICU, some studies have supported the fact that the majority of in-hospital deaths occurred during the first days of ICU admission, while a greater risk of mortality at a later stage was associated with discharge from the hospital, despite the need for mechanical ventilation (22,23). In the present study, it was not demonstrated that LOS in the ICU was a risk factor for mortality.

In a previous study that compared the mortality and survival rates of patients with COVID-19, paraclinical data such as lymphopenia, thrombocytopenia and neutrophilia on admission were the most frequent laboratory findings in the deceased group (24). In the present study, infection with SARS-CoV-2 was positively associated with mortality in the univariate and multivariate analysis.

As regards the prescription of antibiotics, some studies have confirmed that hospitalized patients are administered irrational amounts of antibiotics, and in these cases, a higher mortality rate was documented (25,26). Despite this, in the present study, there was no association between antibiotic prescription and the mortality or survival rates of patients in the ICU, although all patients received antibiotic treatment. The only observation was that orally administered vancomycin was negatively associated with mortality in patients with CPE infection; this treatment was also administered to patients who had an associated *Clostridioides difficile* infection. Nevertheless, from a statistical point of view, further investigations are necessary to determine whether this treatment is a protective factor in the present study group.

Consistent with the results of other studies, it was observed that the predominant CPE strains included the *Klebsiella spp.* group and not *Escherichia coli* (27,28). The ECDC states that In Europe, on average, 1.3 patients per 10.000 hospital admissions had a carbapenemase-producing *Klebsiella pneumoniae* or *Escherichia coli* infection, with the highest incidence found in Southern and Southeastern Europe (29), where Romania is also located.

In Romania, only a limited number of studies have been conducted on CPE strains (5,30). In a previous study conducted in the authors' hospital, the predominance of *Klebsiella* pneumoniae carbapenemase was documented (5) and a multicenter study conducted in eight hospitals in Romania revealed that OXA-48 carbapenemase was the most prevalent carbapenemase during the study period (30).

The present study has some limitations, in that not all the risk factors for mortality in hospitalized patients with CPE infection were investigated, and the fact that although the data were gathered from records spanning over a period of over a period of 4 years (September, 2017 to October, 2021), the number of patients was low.

In conclusion, in the present study, it was observed that the majority of CPE strains were detected in 2021, with most patients presenting bacterial colonization, with no difference in mortality regarding CPE colonization and CPE infection. The LOS in patients who acquired CPE did not influence mortality, while infection with SARS-CoV-2 increased the risk of mortality 16.16-fold, and invasive mechanical ventilation increased the risk of mortality by 14.98-fold.

## Figures and Tables

**Table I:** The CPE strains in patients in the ICU.

Microorganism, n (%)	Cases group (CPE strains and mortality) (n=34) (%)	Control group (CPE strains and survival) (n=30) (%)	P-value^a^
CPE strains			
*Klebsiella spp*.	31 (91.2)	22 (73.4)	0.38
*Escherichia coli*	3 (8.8)	8 (26.7)	0.059
Source of CPE strains			
Rectal swab	27 (79.4)	22 (73.4)	0.56
Urine culture	2 (5.9)	4 (13.4)	0.08
Sputum	8 (23.5)	3(10)	0.15
Blood culture	3 (8.8)	0 (0)	0.09
Skin swab	0 (0)	1 (3.4)	0.28

^a^Data were analyzed using the Chi-squared test. CPE, carbapenemase-producing Enterobacterales; ICU, intensive care unit.

**Table II:** Characteristics of patients with CPE strains admitted to the ICU.

Characteristics of patients with CPE strains, n (%)	Cases group (CPE strains and mortality) (n=34)	Control group (CPE strains and survival) (n=30)	P-value^a^	Univariate analysis OR (95% CI)
Sex, male	24 (70.6%)	21 (70%)	0.95	1.02 (0.35-3.01)
Sex, female	10 (29.4%)	9 (30%)	-	-
Average age, years	63.88	64.67	-	-
≥65 Years old	19 (55.9%)	17 (56.7%)	0.95	0.96 (0.36-2.60)
Charlson Comorbidity Index (CCI) ≥4	18 (52.9%)	16 (53.3%)	0.97	0.98 (0.36-2.63)
Admission with COVID-19	30 (88.2%)	8 (26.7%)	**0.001**	**20.62 (5.50-77.23)**
Admission with a bacterial infection Previous	5 (14.7%)	17 (56.7%)	**0.001**	**0.13 (0.04-0.43)**
hospitalization	7 (20.6%)	13 (43.3%)	0.003	0.15 (0.04-0.56)
Previous antibiotic therapy	22 (64.7%)	14 (46.7%)	0.52	1.57 (0.38-6.43)
Leukopenia on admission	26 (76.5%)	20 (66.7%)	0.38	1.62 (0.54-4.86)
C-reactive protein (CRP) level >5 mg/l at admission	33 (97.1%)	26 (86.7%)	0.12	5.07 (0.53-48.20)
Invasive mechanical ventilation	13 (38.2%)	1 (3.3%)	**0.001**	**17.95 (2.17-148.08)**
CPE colonization	27 (79.4%)	25 (83.3%)	0.68	0.77 (0.21-2.74)
CPE infection	13 (38.2%)	8 (26.7%)	0.32	1.70 (0.58-4.93)
Length of stay in ICU >3 days after CPE diagnosis	29 (85.3%)	24 (80%)	0.57	1.45 (0.39-5.34)
Length of stay in ICU, median (IQR)	9.61	9.46	-	-

^a^Data were analyzed using the Chi-squared test. Values in bold font indicate statistically significant differences (P≤0.05). CPE, carbapenemase-producing Enterobacterales; ICU, intensive care unit; OR, odds ratio, CI, confidence interval.

**Table III:** Treatment of patients admitted to the ICU.

Treatment of patients with CPE strains, n (%)	Cases group (CPE strains and mortality) (n=34) (%)	Control group (CPE strains and survival) (n=30) (%)	P-value^a^	Univariate analysis OR (95% CI)
Antibiotic treatment	34(100)	26 (86.7)	-	-
Reserve antibiotic treatment	26 (76.5)	16 (53.3)	0.052	2.84 (0.97-8.28)
Ceftazidim-avibactam	2 (5.9)	4 (13.3)	0.30	0.40 (0.06-2.39)
Ceftolozane-tazobactam	1 (2.94)	0 (0)	-	-
Imipenem-cilastatin-relebactam	0 (0)	1 (3.3)	-	-
Colistin	5 (14.7)	2 (6.7)	0.30	2.41 (0.43-13.48)
Carbapenem	17(50)	8 (26.7)	0.056	2.75 (0.96-7.87)
Cephalosporins	10 (29.4)	4 (13.3)	0.12	2.70 (0.74-9.79)
Piperacillin-tazobactam	1 (2.9)	1 (3.3)	0.92	0.87 (0.05-14.68)
Quinolones	12 (35.3)	7 (23.3)	0.19	0.38 (0.08-1.70)
Oral vancomycin	1 (2.9)	6(20)	**0.025**	0.12 (0.01-0.97)
Linezolid	8 (23.5)	2 (6.7)	0.06	4.30 (0.83-22.18)
Amikacin	5 (14.7)	5 (16.7)	0.82	0.86 (0.22-3.32)
Doxycycline	4 (11.8)	2 (6.7)	0.48	1.86 (0.31-11.00)
Corticosteroids	28 (82.4)	15(50)	**0.006**	**4.66 (1.49-14.52)**

^a^Data were analyzed using the Chi-squared test. Values in bold font indicate statistically significant differences (P≤0.05). CPE, carbapenemase-producing Enterobacterales; ICU, intensive care unit; OR, odds ratio, CI, confidence interval.

**Table IV:** Independent risk factors for mortality.

			95% CI^a^	
Mortality predictors, multivariate analysis	P-value	OR	Lower	Upper
Admission with COVID-19	**0.001**	16.26	3.56	74.14
Invasive mechanical ventilation	**0.002**	14.98	1.35	166.22
Corticosteroids	0.56	1.56	0.34	7.16

^a^Data were analyzed using logistic regression analysis. Values in bold font indicate statistically significant differences (P≤0.05). OR, odds ratio, CI, confidence interval.

## Data Availability

The data that support the findings of this study are available from the authors, but restrictions apply to the availability of these data, which were used under license for the current study, and so are not publicly available. Data are however available from the authors upon reasonable request and with permission from the Clinical Infection Diseases Hospital, Constanta (NR 1/20/01.2023. CODE F.05.PO.17.00-ACFOCG).
